# Complex phylogeographic history of central African forest elephants and its implications for taxonomy

**DOI:** 10.1186/1471-2148-7-244

**Published:** 2007-12-19

**Authors:** Mireille B Johnson, Stephen L Clifford, Benoît Goossens, Silvester Nyakaana, Bryan Curran, Lee JT White, E Jean Wickings, Michael W Bruford

**Affiliations:** 1 Centre International de Recherches Médicales de Franceville (CIRMF), BP 769, Franceville, Gabon; 2 Cardiff University, Biodiversity and Ecological Processes Group (BEPG), Cathays Park, Cardiff CF10 3TL, Wales, UK; 3 Makerere University, Institute of Environment and Natural Resources (MUIENR), P.O. Box 7298, Kampala, Uganda; 4 Wildlife Conservation Society (WCS), BP 7847, Libreville, Gabon

## Abstract

**Background:**

Previous phylogenetic analyses of African elephants have included limited numbers of forest elephant samples. A large-scale assessment of mitochondrial DNA diversity in forest elephant populations here reveals a more complex evolutionary history in African elephants as a whole than two-taxon models assume.

**Results:**

We analysed hypervariable region 1 of the mitochondrial control region for 71 new central African forest elephants and the mitochondrial cytochrome b gene from 28 new samples and compare these sequences to other African elephant data. We find that central African forest elephant populations fall into at least two lineages and that west African elephants (both forest and savannah) share their mitochondrial history almost exclusively with central African forest elephants. We also find that central African forest populations show lower genetic diversity than those in savannahs, and infer a recent population expansion.

**Conclusion:**

Our data do not support the separation of African elephants into two evolutionary lineages. The demographic history of African elephants seems more complex, with a combination of multiple refugial mitochondrial lineages and recurrent hybridization among them rendering a simple forest/savannah elephant split inapplicable to modern African elephant populations.

## Background

The taxonomic status of the African elephant (*Loxodonta africana*) has been debated since the turn of the 20^th ^century [1] and up to 22 subspecies have been described [2]. However, modern taxonomy refers to two types, with their names reflecting the habitat in which they are found, namely the larger savannah (*Loxodonta africana africana*) (Blumenbach 1797) and the smaller forest (*Loxodonta africana cyclotis*) (Matschie 1900) elephants. It has become increasingly established in the literature that forest and savannah elephants are distinct species (*L. Africana and L. cyclotis*) [3-7], with recent publications considering their datasets in the light of this concept. The most persuasive genetic basis for a two-taxon model originates from a series of studies exploring patterns of differentiation at nuclear loci, culminating in a study using male inherited *Y-chr*, andbi-parentally inherited *X-chr *sequences [6] that concluded "*there was a deep and almost complete separation between African forest and African savannah elephants."* In this study, divergent nuclear DNA sequences segregated with either forest or savannah elephant morphological types. There were, however, a number of exceptions, including a forest elephant from Garamba in the Democratic Republic of Congo (DRC, where forest and savannah populations are sympatric) that had nuclear sequences typical of savannah elephants and two savannah elephants from Cameroon (at the limit of the forest-savannah transition zone) that had nuclear sequences typical of forest elephants [6]. The study estimated the divergence between the savannah and forest elephants to be 3 million years. The two-taxon argument has also been used to explain data from two nuclear microsatellite DNA [5,7] and one morphological study [8,9]. However, subsequently Debruyne [10] performed a morphometric analysis of museum elephant skulls, and found evidence for a continuum between two morphotypes, suggesting that, despite historical separation that promoted subdivision, these two forms freely interbreed wherever their ranges intersect.

Molecular studies using mitochondrial (mt) DNA [10,11] including data from the study by Roca *et al *[6] have pointed to a more complex scenario for African elephants. Debruyne [10] examined several thousand base pairs of mtDNA from elephants across Africa and although he also reported two highly divergent molecular clades, these did not conform to the morphological delineations of *cyclotis *and *africana*. He interpreted these results as a consequence of incomplete isolation between forest and savannah African elephant populations, followed by recurrent and ongoing introgression between the two forms. Roca *et al*. [6] obtained very similar mitochondrial results but explained the non-concordance between mitochondrial and nuclear markers as a result of cytonuclear genomic disassociation such that the mitochondrial tree did not reflect the species tree. The mtDNA results observed were explained as having arisen during episodes of backcrossing between successive generations of savannah males with forest females, leading to half of extant savannah elephants surveyed possessing 'forest' typical mitochondrial haplotypes but almost exclusively 'savannah' nuclear X and Y-chromosomal DNA. Eggert *et al*. [11] (in addition to Nyakaana et al.'s mitochondrial sequences [12]) included samples from west Africa and found a more complex picture using mtDNA and nuclear microsatellites, suggesting that western savannah and forest elephants formed a potential third *Loxodonta *taxonomic unit. Finally, Roca *et al.*[13]recently revisited the question with a statistical re-analysis of eight morphological and genetic datasets (nuclear and mitochondrial) including their own and those of Eggert *et al.*[11] and Debruyne [10] and reconfirmed their initial interpretation of a two taxon model with cyto-nuclear genomic dissociation.

The above-mentioned studies largely share a pronounced lack of forest elephant data. The nuclear DNA studies [4,11] featured limited sampling from central African forest elephants. Despite describing a narrow hybrid zone between the two elephant types, only one population located in this zone (Garamba, (DRC)) was included and none from elsewhere in DRC or from west central Africa were examined. Elsewhere, Debruyne [10] included elephants from across DRC in his study but was again limited by sample size. The study by Eggert *et al*. [11] was limited by the inclusion of only two populations of Central African forest elephants, both from the edge of the forest range in Cameroon which may conceivably have influenced their conclusion of the genetic uniqueness of forest and western elephants. To date, no study has addressed the partitioning of genetic diversity in the equatorial forests of Africa. Further, the potential effect of Pleistocene forest refugia was partially addressed by Eggert *et al.*[11] and also previously reported as having a major influence on large mammal distribution and range dynamics [14-18] has yet to be addressed in African elephants. Here we report results from the most extensive sample of forest elephants to date, from the core of their range, and compare these results with previously published mitchondrial DNA sequences for savannah elephants from east and southern Africa and populations from west Africa and DRC.

We examined the phylogeographic history, population structure and past demography of African elephants using patterns of molecular diversity for the mtDNA control region and cytochrome b gene. Since mtDNA is maternally inherited, this marker provides a female-biased view of population history and structure. We included the most variable mtDNA segment, the hypervariable region 1 (HVR1) of the control region since it has a high rate of nucleotide change, allowing recently diverged lineages to be distinguished [19-21]. This segment is equivalent to data previously published by Eggert *et al*. [11] and Debruyne [10], allowing us to examine forest elephant sequences within the context of a sample set with the largest geographic coverage. We could not use Roca's mtDNA sequences as he studied a different fragment (ND5 instead of control region).

## Results

### Central forest samples

We sequenced 316 bp of HVR1 of the control region from 71 samples and 396 bp of the cytochrome b from 28. No nuclear copies of mitochondrial DNA (*Numts*) were detected for either sequence.

### Combined sequences

#### Genetic diversity

**Figure 1:**
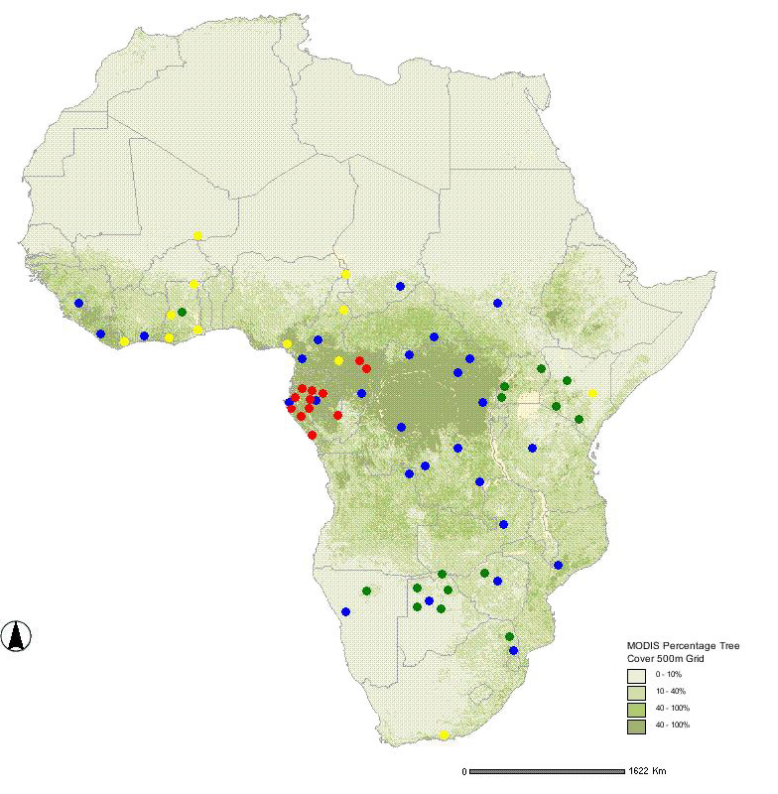
**Map of Africa showing approximate sampling sites from previous mtDNA studies combined with those from this study**. The green, yellow and blue dots are sampling sites from Nyakaana et al. [12], Eggert *et al*. [11] and Debruyne [10], respectively. The red dots are the sites from this study.

For HVR1, we analysed 189 sequences from 66 sites across Africa in both forest and savannah elephants (Figure 1). Of these 102 were from forest elephants (71 samples from the present study and 31 from Genbank) and 87 savannah elephants (all from Genbank). The combined dataset comprised eighty-eight haplotypes (33 and 51 from forest and savannah elephants, respectively) and four haplotypes found in both types. Of the 21 central African forest elephant haplotypes identified in this study, 17 were novel (Genbank accessions EU096114 – EU096130). Mean nucleotide diversity (*π*) for HVR1 sequences for all African elephants was 0.030 (SD = 0.015), while mean haplotype diversity (*h*) was 0.985 (SD = 0.003). When haplotypes were divided into forest and savannah, based on prior designation, the forest population *π *was 0.022 (SD = 0.11), significantly lower than for savannah elephants (0.034, SD = 0.017; *p *< 0.001). The mean haplotype diversity for forest and savannah populations was 0.960 (SD = 0.007) and 0.986 (SD = 0.004), respectively. The lowest nucleotide diversity of all groupings was for the new central African forest samples in this study (0.013, SD = 0.007), while haplotype diversity was 0.947 (SD = 0.009).

For cytochrome *b*, 100 sequences were analysed, 28 from this study, 27 provided by SN and 45 from Genbank. Forty-four haplotypes were identified including three and 22 new forest and savannah elephant sequences, respectively (Genbank accessions EU115995 – EU116019). Of the 44 haplotypes, 32 were found in savannah elephants and 10 in forest elephants, with two haplotypes found in both. Mean *π *for cytochrome *b *was 0.023 (0.012) for all elephants. When forest and savannah elephants were subdivided, *π *was again significantly lower for forest populations (0.009, SD = 0.005) than for savannah populations (0.026, SD = 0.013; *p *< 0.001). These results contrast with the study of Roca *et al*. (2005) who reported 15 haplotypes for 281 elephants at the mitochondrial ND5 locus and described low genetic diversity as being typical for savannah elephants.

#### Population structure

**Figure 2:**
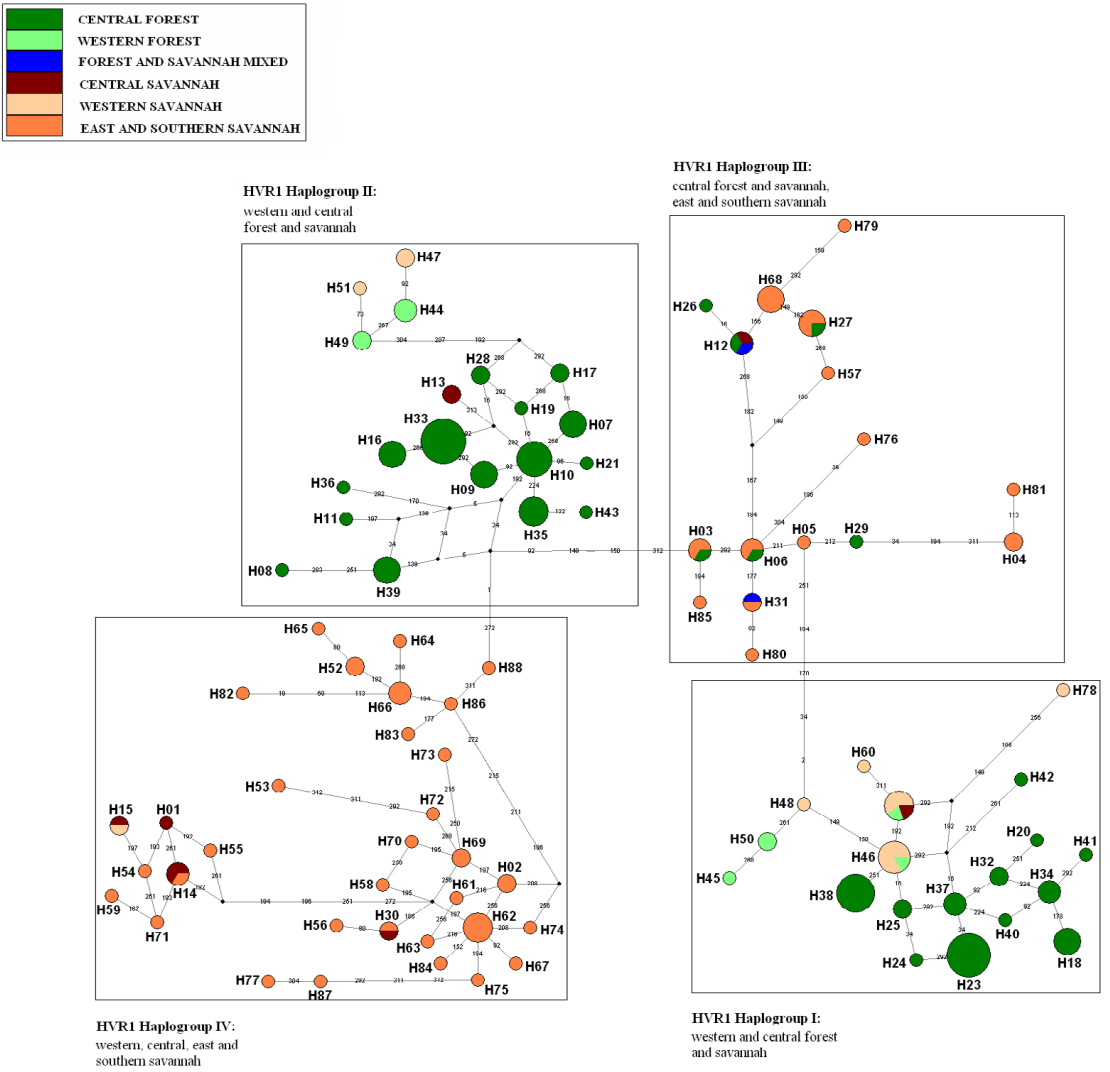
**Median-joining networks for African elephants HVR1 mtDNA haplotypes**. Circle size is proportional to the haplotype frequency. The numbers on the connecting line determine the number of substitutions estimated by NETWORK V.4. 1. 1. 1. The entire list of haplotypes for HVR1 MJN can be found in **Table 1**.

**Figure 3:**
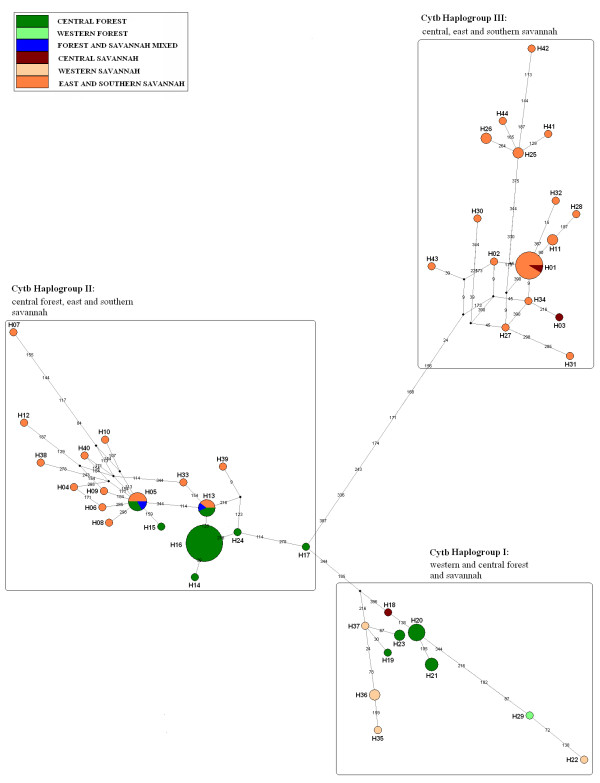
**Median-joining networks for African elephants cytochrome *b *mtDNA haplotypes**. Circle size is proportional to the haplotype frequency. The numbers on the connecting line determine the number of substitutions estimated by NETWORK V.4. 1. 1. 1. The entire list of haplotypes for cytochrome *b *MJN can be found in **Table 2**.

The median joining networks for the HVR1 and cytochrome *b *sequences (Figures 2 and 3, respectively), exhibit patterns consistent with a complex demographic history. The HVR1 pattern is more complex (comprising four haplogroups – here labelled HVR1 Haplogroup I, II, III and IV) than for cytochrome *b *(three haplogroups -labelled Cytb Haplogroup I, II and III). Haplotype designations for this and previous studies for both sequences are found in Table 1 (HVR) and Table 2 (cyt b). For the HVR1 region, the most obvious feature is that central African forest elephants (excluding those from DRC) fall into two separate groups (HVR1 Haplogroups I and II) with little geographic structuring, consisting of 19 (HVR1 Haplogroup I) and 20 (HVR1 Haplogroup II) haplotypes with variable frequencies. Only two forest elephants from DRC, share the same haplotype with other forest elephants in HVR1 Haplogroup II. The remaining seven DRC forest elephant haplotypes (all south-east of the Congo River), group with sequences in HVR1 Haplogroup III (which additionally comprises savannah elephants from eastern and southern Africa and one savannah elephant from Cameroon). The other striking feature is that for West African elephants (from Eggert et al 2002, see Table 1 for haplotype designations), both forest and savannah types possess haplotypes found almost exclusively within the same haplogroup as central African forest elephants (HVR1 Haplogroups I and II). Twenty-five out of 26 haplotypes from west Africa are more closely related to central Forest elephants from Gabon, Congo and CAR. A single western savannah sequence (H15) can be found in HVR1 Haplogroup IV grouping with savannah elephants from eastern, southern and central Africa. Analysis of Molecular Variance (AMOVA) of HVR1 sequences revealed a non-significant (p = 0.065) genetic structure (18.62% variation among populations) when populations were grouped according to geographic distribution (west, central, east and southern Africa).

**Table 1:** HVR1 haplotypes used in the Figure 2. Haplotype frequency is indicated in brackets when there is more than one.

Haplotype	**Taxon**	Designation*	Geographic origin	Genbank accession numbers	Author
H01	*Loxodonta africana africana*	Angola1	Angola	AY741072	Debruyne 2005
H02 (2)	*L. a. africana*	Botswana1, BOT4	Botswana	AY741074, AF106230	Debruyne 2005, Nyakaana *et al*. 2002
H03 (3)	*L. a. africana, L. a. cyclotis*	BOT2, BOT21, DRC4	Botswana, DRC	AF106228, AF106234, AY359275	Nyakaana *et al*. 2002, Debruyne 2005
H04 (2)	*L. a. africana*	BOT9, Zimbabwe2	Botswana, Zimbabwe	AF106231, AY741329	Nyakaana *et al*. 2002, Debruyne 2005
H05	*L. a. africana*	BOT15	Botswana	AF106232	Nyakaana *et al*. 2002
H06 (3)	*L. a. africana, L. a. cyclotis*	BOT16, DRC1, Zimbabwe4	Botswana, DRC, Zimbabwe	AF106233, AY359277, AY742799	Nyakaana *et al*. 2002, Debruyne 2005
H07 (4)	*L. a. cyclotis*	Bmbo6, Dja39, CAR3214, CAR394	Cameroon, CAR	AF527653, AF527647	Eggert *et al*. 2002, this study
H08	*L. a. cyclotis*	Cameroon1	Cameroon	AY359267	Debruyne 2005
H09 (4)	*L. a. cyclotis*	Cameroon2, Bmbo1, Bmbo37, NYO0310	Cameroon, Gabon	AY359269, AF527646, AF527649	Debruyne 2005, Eggert *et al*. 2002, this study
H10 (7)	*L. a. cyclotis*	Bmbo16, Bmbo43, CAR274, CAR297, Congo2, NN0713, NN2911	Cameroon, CAR, CR	AF527648, AF527650, AY359268	Eggert *et al*. 2002, Debruyne 2005, this study
H11	*L. a. cyclotis*	Dja34	Cameroon	AF527651	Eggert *et al*. 2002
H12 (3)	*L. africana, L.a. fricana*, *L. a. cyclotis*	DRC13**, B1, DRC9	DRC, Cameroon	AY741081, AY359279, AF527654,	Debruyne 2005, Eggert *et al*. 2002
H13 (2)	*L. a. africana*	B7, Waza15	Cameroon	AF527655, AF527659	Eggert *et al*. 2002
H14 (3)	*L. a. africana*	B8, Waza10, Sudan1	Cameroon, Sudan	AF527656, AF527658, AY741073	Eggert *et al*. 2002, Debruyne 2005
H15 (2)	*L. a. africana*	Waza27, Mali2	Cameroon, Mali	AF527660, AF527666	Eggert *et al*. 2002
H16 (4)	*L. a. cyclotis*	CAR3622, NN059, NN279, NN3014	CAR, CR		This study
H17 (2)	*L. a. cyclotis*	CAR3315, CAR381	CAR		This study
H18 (4)	*L. a. cyclotis*	CAR5712, AFE82lan, MDC012, NOG053,	CAR, Gabon		This study
H19	*L. a. cyclotis*	CAR1	CAR	AY359272	Debruyne 2005
H20	*L. a. cyclotis*	CAR309	CAR		This study
H21	*L. a. cyclotis*	CAR3519	CAR		This study
H22 (5)	*L. a. africana*	Chad1, K68, RVV15, Mole13, WA6	Chad, Ghana	AY741080, AF527643, AF527641, AF527676, AF106243	Eggert *et al*. 2002, Debruyne 2005, Nyakaana *et al*. 2002
H23 (10)	*L. a. cyclotis*	NN3218, Lan027, LOP067, LOP51a14, NOG014, NOG025, NOG026, Mpa01, Mpa028, RAB0113	RC, Gabon		This study
H24	*L. a. cyclotis*	Congo1	RC	AY359266	Debruyne 2005
H25 (2)	*L. a. cyclotis*	CKT04a14, RAB275	RC, Gabon		This study
H26	*L. a. cyclotis*	DRC2	DRC	AY359270	Debruyne 2005
H27 (4)	*L. a. africana *and *L. a. cyclotis*	KV8, MF1, MF5, DRC3	Uganda, DRC	AF106206, AF106209, AF106210, AY359271	Nyakaana *et al*. 2002, Debruyne 2005
H28 (2)	*L. a. cyclotis*	DRC6, DRC8	DRC	AY359273, AY359274	Debruyne 2005
H29	*L. a. cyclotis*	DRC5	DRC	AY359276	Debruyne 2005
H30 (2)	*L. a. africana*	DRC11, AM1	DRC, Kenya	AY741078, AF106217	Nyakaana *et al*. 2002, Debruyne 2005
H31 (2)	*L. africana *and *L. a. africana*	DRC17**, QE13	DRC, Uganda	AY742802, AF106213	Nyakaana *et al*. 2002, Debruyne 2005
H32 (2)	*L. a. cyclotis*	IVI1011, RAB067	Gabon		This study
H33 (11)	*L. a. cyclotis*	Igl032, AFE85Igl, AFE86Igl, AFE88Igl, IVI1012, IVI043, LOA0310, LOP146, Mpa0319, RAB0215, WAK0410	Gabon		This study
H34 (3)	*L. a. cyclotis*	Lan015, Lan15911, RAB131	Gabon		This study
H35 (5)	*L. a. cyclotis*	Lan1566, IVI05a6, IVI05b8, RAB032, WAK0817	Gabon		This study
H36	*L. a. cyclotis*	Lan16014	Gabon		This study
H37 (3)	*L. a. cyclotis*	Gabon2, LOP0710, PBA023	Gabon	AY359265	Debruyne 2005, this study
H38 (8)	*L. a. cyclotis*	IVI06b2, Kes0721, Kes0819, LOA068, AFE79LOP, PBA0510, RAB044, RAB1118	Gabon		This study
H39 (4)	*L. a. cyclotis*	Kes0211, Kes0314, Kes0517, PBA0714	Gabon		This study
H40	*L. a. cyclotis*	Gabon1	Gabon	AY359278	Debruyne 2005
H41	*L. a. cyclotis*	NOG0810	Gabon		This study
H42	*L. a. cyclotis*	PBA0612	Gabon		This study
H43	*L. a. cyclotis*	IVI05a5	Gabon		This study
H44 (3)	*L. a. cyclotis*	Bia3, Bia69, Liberia1	Ghana, Liberia	AF527677, AF527680, AY741079	Eggert *et al *2002, Debruyne 2005
H45	*L. a. cyclotis*	Bia48	Ghana	AF527678	Eggert *et al *2002
H46 (6)	*L. a. cyclotis *and *L. a. africana*	Bia64, RVV22, Mole9, WA3, WA14, Mali7	Ghana, Mali	AF527679, AF527642, AF527675, AF106242, AF106245, AF527667	Eggert *et al *2002, Nyakaana *et al *2002
H47 (2)	**L. a. africana**	Mole3, Mali14	Ghana, Mali	AF527674, AF527668	Eggert *et al *2002
H48	*L. a. africana*	Mole33	Ghana	AF527683	Eggert *et al *2002
H49 (2)	*L. a. cyclotis*	Tai6, Tai17	Ivory Coast	AF527670, AF527671	Eggert *et al *2002
H50 (2)	*L. a. cyclotis*	Tai19, Tai29	Ivory Coast	AF527672, AF527673	Eggert *et al *2002
H51	**L. a. africana**	IvoryCoast1	Ivory Coast	AY741327	Debruyne 2005
H52 (2)	*L. a. africana*	SouthAfrica3, Zimbabwe1	South Africa, Zimbabwe	AY741320, AY741321	Debruyne 2005
H53	*L. a. africana*	MM4	Kenya	AF106214	Nyakaana *et al *2002
H54	*L. a. africana*	MM19	Kenya	AF106215	Nyakaana *et al *2002
H55	*L. a. africana*	MM20	Kenya	AF106216	Nyakaana *et al *2002
H56	*L. a. africana*	AM2	Kenya	AF106218	Nyakaana *et al *2002
H57	*L. a. africana*	AM10	Kenya	AF106219	Nyakaana *et al *2002
H58	*L. a. africana*	AM12	Kenya	AF106220	Nyakaana *et al *2002
H59	*L. a. africana*	SA8	Kenya	AF106221	Nyakaana *et al *2002
H60	*L. a. africana*	Mali28	Mali	AF527669	Eggert *et al *2002
H61	*L. a. africana*	Mozambique1	Mozambic	AY741076	Debruyne 2005
H62 (5)	*L. a. africana*	Namibia1, Addo5, Uganda1, QE1, Zimbabwe10	Namibia, South Africa, Uganda, Zimbabwe	AY741325, AF527682, AF106211, AY741323, AY742800	Nyakaana *et al *2002, Eggert *et al *2002, Debruyne 2005
H63	*L. a. africana*	KH2	Namibia	AF106239	Nyakaana *et al *2002
H64	*L. a. africana*	Addo1	South Africa	AF527681	Eggert *et al *2002
H65	*L. a. africana*	KG1	South Africa	AF106240	Nyakaana *et al *2002
H66 (3)	*L. a. africana*	KG2, Tanzania2, Zimbabwe7	South Africa, Tanzania, Zimbabwe	AF106241, AY741070, AY741067	Nyakaana *et al *2002, Debruyne 2005
H67	*L. a. africana*	Tanzania1	Tanzania	AY742801	Debruyne 2005
H68 (4)	*L. a. africana*	QE4, Zambia1, Af9, Af10	Uganda, Zambia, Kenya	AF106212, AY741328, AF527639, AF527640	Nyakaana *et al *2002, Eggert *et al *2002, Debruyne 2005
H69 (2)	*L. a. africana*	Uganda2, KV1	Uganda	AY741077, AF106203	Nyakaana *et al *2002, Debruyne 2005
H70	*L. a. africana*	KV2	Uganda	AF106204	Nyakaana *et al *2002
H71	*L. a. africana*	KV7	Uganda	AF106205	Nyakaana *et al *2002
H72	*L. a. africana*	KV17	Uganda	AF106207	Nyakaana *et al *2002
H73	*L. a. africana*	KV28	Uganda	AF106208	Nyakaana *et al *2002
H74	*L. a. africana*	WC2	Namibia	AF106235	Nyakaana *et al *2002
H75	*L. a. africana*	WC4	Namibia	AF106236	Nyakaana *et al *2002
H76	*L. a. africana*	WC6	Namibia	AF106237	Nyakaana *et al *2002
H77	*L. a. africana*	WC13	Namibia	AF106238	Nyakaana *et al *2002
H78	*L. a. africana*	WA11	Ghana	AF106244	Nyakaana *et al *2002
H79	*L. a. africana*	AF8	Kenya	AF527638	Eggert *et al *2002
H80	*L. a. africana*	ZBE1	Zimbabwe	AF106222	Nyakaana *et al*. 2002
H81	*L. a. africana*	ZBE2	Zimbabwe	AF106223	Nyakaana *et al*. 2002
H82	*L. a. africana*	ZBE3	Zimbabwe	AF106224	Nyakaana *et al*. 2002
H83	*L. a. africana*	ZBE4	Zimbabwe	AF106225	Nyakaana *et al*. 2002
H84	*L. a. africana*	ZBE5	Zimbabwe	AF106226	Nyakaana *et al*. 2002
H85	*L. a. africana*	ZBE6	Zimbabwe	AF106227	Nyakaana *et al*. 2002
H86	*L. a. africana*	Zimbabwe3	Zimbabwe	AY741069	Debruyne 2005
H87	*L. a. africana*	Zimbabwe6	Zimbabwe	AY741071	Debruyne 2005
H88	*L. a. africana*	Zimbabwe5	Zimbabwe	AY741322	Debruyne 2005

* Original name from each author (Debruyne, 2005; Eggert *et al*. 2002; Nyakaana *et al*. 2002; and this study. ** Sample sharing both, forest and savannah haplotypes, according to the author (Debruyne, 2005).

**Table 2:** cytochrome *b *haplotypes used in Figure 3. Haplotype frequency is indicated in brackets when there is more than one.

Haplotype	**Taxon**	Designation*	Geographic origin	Genbank accession numbers	Author
H01 (12)	*L. a. africana*	AM1, AM2, QE51, WC4, BO1, DRC11, MO1, NA1, TA1, UG1, UG3, ZI10	Kenya, Uganda, Namibia, Botswana, DRC, Mozambique, Tanzania, Zimbabwe	AY741074, AY741078, AY741076, AY741325, AY742801, AY741323, AY741324, AY742800	SN, Debruyne 2005
H02	*L. a. africana*	AM12	Kenya		SN
H03	*L. a. africana*	AN1	Angola	AY741072	Debruyne 2005
H04	*L. a. africana*	BOT13	Botswana		SN
H05 (6)	*L. a. cyclotis, L.a. africana, L. africana*	DRC1, DRC4, DRC17**, BOT17, ZI2, ZI4	DRC, Botswana, Zimbabwe	AY359275, AY359277, AY742802, AY741329, AY742799	Debruyne 2005, SN
H06	*L. a. africana*	BOT18	Botswana		SN
H07	*L. a. africana*	BOT1	Botswana		SN
H08	*L. a. africana*	BOT21	Botswana		SN
H09	*L. a. africana*	BOT25	Botswana		SN
H10	*L. a. africana*	BOT2	Botswana		SN
H11 (2)	*L.a. africana*	BOT4, ET1	Botswana		SN
H12	*L.a. africana*	BOT9	Botswana		SN
H13 (5)	*L. a. cyclotis, L.a. africana, L. africana*	DRC2, DRC9, DRC13**, KV8, MF5	DRC, Uganda	AY359270, AY359279, AY741081	Debruyne 2005, SN
H14	*L. a. cyclotis*	DRC3	DRC	AY359271	Debruyne 2005
H15	*L. a. cyclotis*	DRC5	DRC	AY359276	Debruyne 2005
H16 (22)	*Loxodonta africana cyclotis*	DRC6, DRC8, Cameroon2, CAR1, Congo2, CAR274, CAR297, CAR3315, CAR3417, CAR405, CAR3723, CAR4311, IVI1012, KES0819, LOP146, NN0713, NN232, NN267, NN279, NN2911, NN3116, NN3218	DRC, Cameroon, CAR, RC, Gabon	AY359268, AY359269, AY359272, AY359273, AY359274	Debruyne 2005, MJ
H17	*L. a. cyclotis*	Cameroon1	Cameroon	AY359267	Debruyne 2005
H18	*L.a. africana*	Chad1	Chad	AY741080	Debruyne 2005
H19	*L. a. cyclotis*	CKT04a14	RC		MJ
H20 (5)	*L. a. cyclotis*	Congo1, MPA01, MPA02, NOG014, NOG026	RC, Gabon	AY359266	Debruyne 2005, MJ
H21 (3)	*L. a. cyclotis*	Gabon2, Gabon1, NN255	Gabon, RC	AY359265, AY359278	Debruyne 2005, MJ
H22	*L.a. africana*	Ivory Coast1	Ivory Coast	AY741327	Debruyne 2005
H23 (2)	*L. a. cyclotis*	IVI06c4, LOPAFE79	Gabon		MJ
H24	*L. a. cyclotis*	KES0314	Gabon		MJ
H25 (2)	*L.a. africana*	Zi5, KG1	Zimbabwe, South Africa	AY741322	SN, Debruyne 2005
H26 (2)	*L.a. africana*	KG2, SouthAfrica3	South Africa	AY741320	Debruyne 2005, SN
H27	*L.a. africana*	KV19	Uganda		SN
H28	*L.a. africana*	KV2	Uganda		SN
H29	*L. a. cyclotis*	Liberia1	Liberia	AY741079	Debruyne 2005
H30	*L.a. africana*	MM19	Kenya		SN
H31	*L.a. africana*	MM20	Kenya		SN
H32	*L.a. africana*	Namibia2	Namibia	AY741326	Debruyne 2005
H33	*L.a. africana*	QE48	uganda		SN
H34	*L.a. africana*	Sudan1	Sudan	AY741073	Debruyne 2005
H35	*L.a. africana*	WA13	Ghana		SN
H36 (2)	*L.a. africana*	WA14, WA15	Ghana		SN
H37	*L.a. africana*	WA6	Ghana		SN
H38	*L.a. africana*	WC6	Namibia		SN
H39	*L.a. africana*	Zambia1	Zambia	AY741328	Debruyne 2005
H40	*L.a. africana*	ZBE1	Zimbabwe		SN
H41	*L.a. africana*	ZBE3	Zimbabwe		SN
H42	*L.a. africana*	ZBE4	Zimbabwe		SN
H43	*L.a. africana*	ZBE5	Zimbabwe		SN
H44	*L.a. africana*	Zimbabwe1	Zimbabwe	AY741321	Debruyne 2005

* Original name from each author (Debruyne, 2005; this study SN = Silvester Nyakaana and MJ = Mireille Johnson) ** Sample sharing both, forest and savannah haplotypes, according to the author (Debruyne, 2005).

As expected, Cytochrome *b *is less variable than HVR1. However, direct comparison between patterns obtained from both regions is compromised here due to a lack of equivalent individuals examined at both loci, specifically for savannah elephants. However the overall pattern when individuals from different populations were examined is consistent with the pattern obtained with HVR1, despite the resolution of only three haplogroups as opposed to four. Savannah elephant haplotypes fall into two distinct haplogroups (Cyt b Haplogroup II and III) as do forest elephant haplotypes (Cyt b Haplogroups I and II). Cytochrome b Haplogroup II, which is divided into two haplogroups for HVR1, is characterised by a network structure in which forest and savannah elephant samples are not overlaid (see Figure 3). Again all western elephants, both forest and savannah, cluster with central African forest elephants (Cyt b Haplogroup I).

#### Demographic history

**Figure 4:**
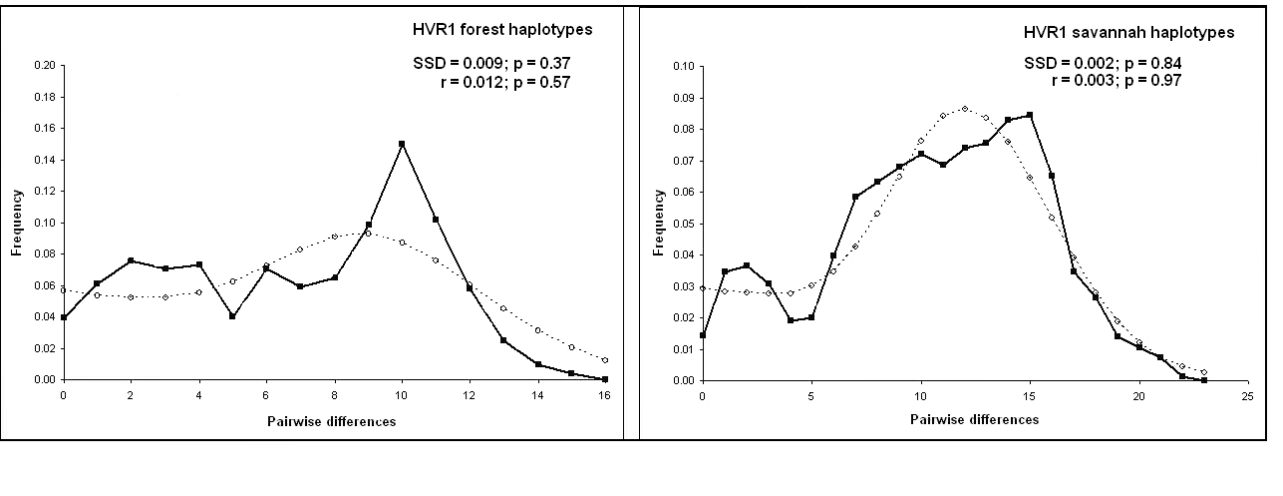
Mismatch distribution of the HVR1 forest and savannah African elephants haplotypes.

**Figure 5:**
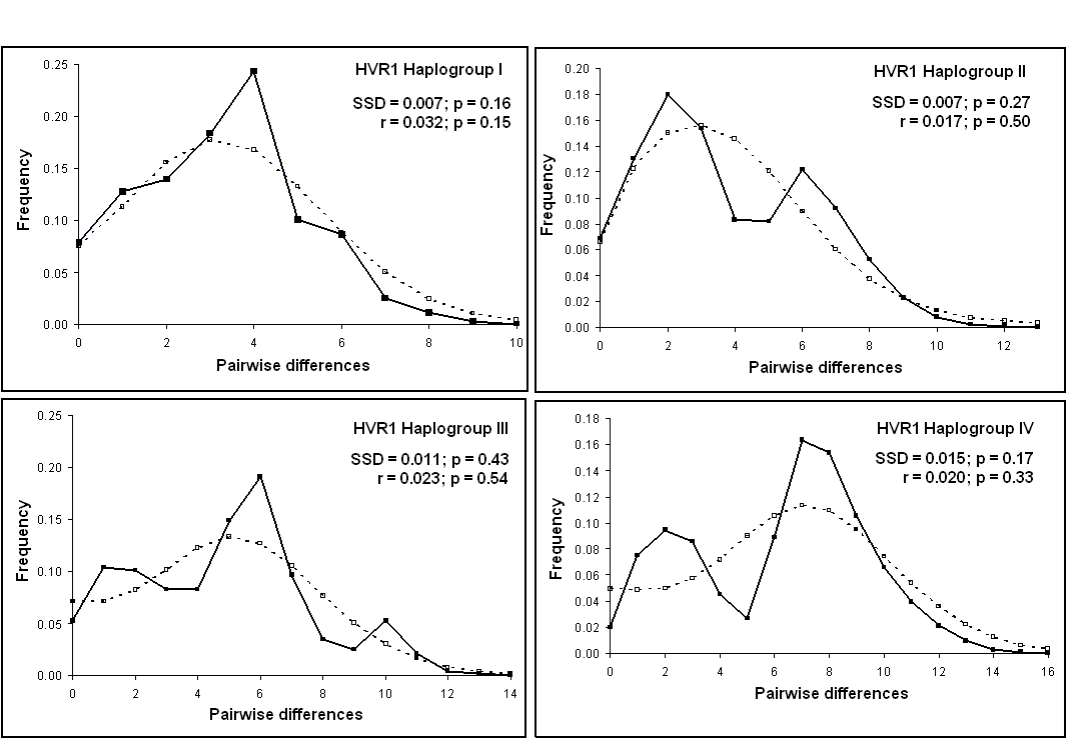
Mismatch distribution of the HVR1 haplogroups of African elephants.

When HVR1 sequences from forest and savannah elephants were examined separately, Fu's *Fs *was -14.2954 (*P *= 0.0021) and -24.4427 (*P *< 0.0001), respectively. Although significant values can indicate historical population expansion, the multimodal pattern (Figure 4) for the forest elephant groups suggests that these populations encompass several subgroups as indicated in the networks. When we examined each haplogroup separately for signatures of demographic change (Table 3), a smooth and predominantly unimodal pattern was observed for HVR1 Haplogroup I, indicating a recent demographic expansion (Figure 5), while HVR1 Haplogroups II, III and IV were more complex, including the presence of some divergent haplotypes.

**Table 3:** Indicators of demographic change.

	Haplogroup I	Haplogroup II	Haplogroup III	Haplogroup IV
Fu's Fs	-7.30	-6.34	-4.61	-22.44
*p*-value	0.006	0.015	0.034	< 0.0001

## Discussion

In the light of the results obtained with the mitochondrial sequences used here, additional interpretations of the history of African elephants become evident and suggest that the conclusions drawn in previous studies may have been hampered by incomplete sample sets. Forest elephants have been affected by cyclical climatic changes that occurred over the last 2.6 million years as the colder drier periods experienced during Pleistocene glacial maxima are believed to have led to the repeated retraction of forest cover into refugial zones followed by re-expansion, fostering allopatric divergence between isolated populations [22] and secondary contact. The forest elephant range is therefore likely to have become centred around such refugia on several occasions. The dataset presented here raises the possibility of at least two different refugia in the central African region harbouring distinct elephant populations that diverged allopatrically. If this was the case, forest elephants possessing distinct mitochondrial genotypes are likely to have come into contact relatively rapidly after the end of the last glaciation (12,000 years BP), when the forests re-expanded [23]. Such a scenario might explain not only the two haplogroups present in forest elephants but also the lower nucleotide diversity that characterises elephant populations found in forest habitat.

This scenario might also explain the high microsatellite diversity reported for forest elephants [5]. If several populations diverged in isolation, accumulating different microsatellite profiles, and subsequently became sympatric as the forest expanded, the large single population that today comprises two central African forest elephant lineages might be expected to have engendered higher microsatellite diversity. Savannah populations, especially those in the south and east, would not have been affected by forest expansion since these areas remained unforested and thus habitat would not have been lost. Those savannah populations that may have been affected are those that may have occurred in areas that subsequently became forested. One explanation for the close genetic proximity between forest and savannah genotypes in DRC could be introgression between savannah haplotypes into forest genomes as forests expanded and savannah habitat was lost. Such introgression would be in the opposite direction to that proposed by Roca *et al. *[4,6].

The results obtained for elephants in west and central Africa have strong implications for the division of elephants into forest and savannah species. These elephants are taxonomically indeterminate [24] and have been described as having an intermediate morphology [8]. Mitochondrially, West African elephants are found in the same haplogroups as the (two) forest elephant lineages of central Africa. If ancient female-mediated introgression between the two forms followed by backcrossing into savannah populations is the reason why western savannah elephants possess largely 'forest' haplotypes then nuclear markers at these loci should resemble predominantly those of southern and eastern savannah elephants today. Alternatively these elephant populations could be an example of protracted gene flow between two forms of elephant, which is ongoing (or was until recently) and that west African savannah elephants are not distinguishable at the genetic or morphological level from their forest counterparts (thus undermining the two-taxon model). A third explanation could be a 'second movement' of elephants out of the forest (from either west or central Africa) and into the savannah. There are insufficient data to determine whether there was a single movement from forest to savannah habitat or whether these were multiple events, precipitating the morphological changes observed today. Whatever the origin of the two types, our data would support continued extensive hybridisation between the two proposed forms.

## Conclusion

Our mitochondrial analysis does not support the simple separation of modern African elephants into two groups. The evidence is most clear in west Africa where savannah elephants are indistinguishable at both the mitochondrial and morphological level from their forest counterparts. The two species model cannot be easily applied in this region and neither do west African elephants represent a third distinct entity. Central African elephant populations west of the Congo river also question the current classification. Forest elephants fall into two major groupings with mitochondrial DNA. Previous studies found two major groups for all African elephants, savannah and savannah/forest perhaps suggesting ancient introgression between forest females and savannah males in the past. However the inclusion of a larger central forest sample in this study would suggest that this explanation is too simple and that African elephants were subject to a more complex demographic history. Phylogenetic and phylogeographic reanalysis of species is important for many reasons but with the massive extinction of species in the wild in the last 50 years accurate descriptions are essential for management of wild resources. For elephants, the classification of species into savannah and forest may not reflect their evolutionary history but simply the habitat in which they currently exist. While ecotypic differentiation has been shown to be the predominant factor driving molecular divergence in one widely distributed African herbivore recently [25], this may not apply in elephants and if it does, may not conform to a simple forest *versus *savannah habitat driven divergence. To develop management strategies incorporating a simple forest/savannah model could be misleading until further lines of evidence give us a clearer picture of the origins and current conservation needs of elephants populations throughout the continent. Future studies should analyse nuclear DNA markers, including those which evolve rapidly, across the range of forest and savannah elephants and especially in transition zones to investigate this complex ongoing process further.

## Methods

### Sampling and laboratory procedures

Elephant sequences from 66 sites across Africa were incorporated (Figure 1). New forest elephant samples (HVR1 mtDNA: n = 71; Cyt *b *mtDNA: n = 28) were obtained using feces from 12 sites in the central African forest block (red dots, Figure 1).

Samples were stored in RNAlater (Ambion RNA *later*^® ^and Qiagen RNA later ™) or silica gel, and DNA was extracted from these using the Qiagen DNA stool mini kit (Qiagen, Hilden, Germany) kit following the manufacturer's protocol.

An approximately 630 bp fragment of mitochondrial DNA was amplified, encompassing the 3' end of the cytochrome *b *gene, transfer RNAs (Threonine, Proline) and 358 bp of the control region. The control region section was amplified in 71 samples using primers MDL3 and MDL5 [26]. Primers AFDL1 and AFDL2 (situated 400 bp from the 3'end of the cytochrome *b *gene through to the 5' end of the control region), and AFDL3 and AFDL4 (situated 377 bp from the 3' end of tRNA proline to the 5'end of the control region) were employed to gain overlapping sequence for some degraded samples [11]. A 494 bp fragment of cytochrome *b *was analysed separately with 28 sequences using the primers L15024 and H15516 [3]. Amplifications were performed in 50 μl containing 50 mM KCl, 10 mM Tris-HCl, 1.5 mM Mg^2+^, 200 μmol of each dNTP, 0.2 μmol of each primer, 1.5 U *Taq *DNA polymerase (Qiagen) and approx. 10 ng of genomic DNA. Thirty to 40 cycles were carried out using a Perkin-Elmer Cetus 9600 or 9700 DNA thermocycler with denaturation at 94°C for 45s, annealing at 63°C for 45s, and extension at 72°C for 45s. PCR products were purified using the Qiagen PCR purification kit and subsequently sequenced commercially (Macrogen, Korea).

### Analysis of genetic diversity and differentiation

Forward and reverse sequences for each individual and the consensus sequences for all individuals were aligned using SEQUENCHER (Gene Codes Corporation 1998, version 3.1.1) and rechecked by eye. Genetic diversity for all geographic locations was estimated using haplotype *h *and nucleotide *π *diversities as implemented in Arlequin ver. 3.0 [27]. Paired t-tests were carried out to assess whether there was significant difference in nucleotide diversity between forest and savannah elephants. Genetic differentiation between pairs of populations was tested using the exact test using 10,000 Markov chain steps, as implemented in ARLEQUIN ver. 3.0, and this program was also employed for nested analysis of molecular variance (AMOVA) to test for patterns of spatial genetic structure. The dataset was divided in forest and savannah groupings and then four regional populations were defined (west, central, east and south). Using AMOVA the correlation among genotype distances is used as an *F*-statistic analog (Phi) at various hierarchical levels.

Weighted maximum likelihood distances [28] were used to derive a median joining network (MJN) with the program NETWORK V4.1.1.1. Haplotype networks may more effectively portray the relationships among sequences for populations than maximum likelihood or maximum parsimony which are the traditional methods developed to define interspecific relationships, leading to poor resolution at the population level [29].

### Analysis of population demography

Tests were performed to detect evidence of past demographic change. We used ARLEQUIN ver.3.0 to perform a pairwise mismatch distribution, comparing the distribution of the observed pairwise nucleotide site differences with the expected distribution in an expanding population [30]. In a single origin, demographically expanding population, mismatches should follow a unimodal Poisson distribution whereas in populations at demographic equilibrium or with sub-groups, the distribution is usually multimodal. We tested the goodness-of-fit of the observed data to a simulated model of expansion with the sum of square deviations (SSD) and the Harpending's raggedness index *r*, using ARLEQUIN.

Population history was also inferred using Fu's *F*_S _test of neutrality [31] as implemented in ARLEQUIN. We chose this test because it is the most powerful coalescent-based neutrality test for detecting population growth for larger sample sizes.

## Authors' contributions

MBJ carried out the molecular genetic studies, analyzed the data and drafted the manuscript as part of her PhD dissertation.

SLC made substantive contributions data analysis and interpretation and helped to draft the manuscript.

SN provided cytochrome *b *sequences for savannah elephants from Ghana and provided comments on the manuscript.

BC and LJTW participated in the design of the study.

BG, EJW and MWB conceived and initiated the study, participated in its coordination, advised on data analysis and helped to draft the manuscript and revise it critically. MWB made substantial text contributions, especially during the review process.

All authors read and approved the final manuscript.
